# Volumetric evaluation of ^99m^Tc-pyrophosphate SPECT/CT for transthyretin cardiac amyloidosis: Methodology and correlation with cardiac functional parameters

**DOI:** 10.1007/s12350-021-02857-7

**Published:** 2021-12-14

**Authors:** Satoru Watanabe, Kenichi Nakajima, Hiroshi Wakabayashi, Hiroto Yoneyama, Shohei Yoshida, Junji Komatsu, Takahiro Konishi, Anri Inaki, Seigo Kinuya

**Affiliations:** 1 Department of Functional Imaging and Artificial Intelligence, Kanazawa University, Kanazawa, Japan; 2 Department of Nuclear Medicine, Kanazawa University Hospital, 13-1 Takara-machi, Kanazawa, 920-8641 Japan; 3 Department of Radiology, Kanazawa University Hospital, Kanazawa, Japan; 4 Department of Cardiovascular Medicine, Kanazawa University Graduate School of Medical Sciences, Kanazawa, Japan; 5 Department of Neurology and Neurobiology of Aging, Kanazawa University Graduate School of Medical Sciences, Kanazawa, Japan

**Keywords:** Amyloid heart disease, SPECT, Image analysis, Hybrid imaging, Cardiomyopathy

## Abstract

**Background:**

Volumetric evaluation of ^99m^Technetium-pyrophosphate (^99m^Tc-PYP) SPECT/CT is a useful method for assessing transthyretin cardiac amyloidosis (ATTR-CA). We investigated the methodology and assessed its relationship with conventional parameters.

**Methods and Results:**

We retrospectively evaluated ^99m^Tc-PYP SPECT/CT scans of 25 patients who underwent endomyocardial biopsy and/or gene testing. Fourteen (56%) patients were diagnosed with ATTR-CA. SPECT/CT images were acquired at 3 hours after injection. Total volumes of the myocardial regions where uptakes were > 1.2 and 1.4 × aortic blood pool SUVmax were evaluated and defined as cardiac pyrophosphate volume (CPV1.2 and CPV1.4). The heart-to-contralateral lung (H/CL) ratio and myocardial SUVmax were also calculated. CPV1.2 achieved the highest sensitivity and specificity in diagnosing ATTR-CA. In patients diagnosed with ATTR-CA (*n* = 14), CPV1.2 negatively correlated with left ventricular ejection fraction and positively correlated with left ventricular posterior wall thickness and QRS duration. The correlation was stronger in CPV1.2 than in the H/CL ratio and SUVmax.

**Conclusion:**

Volumetric evaluation of ^99m^Tc-PYP SPECT/CT may be superior to the H/CL ratio and SUVmax in assessing the disease burden of ATTR-CA. Larger studies are warranted to clarify whether volumetric measurement can assess prognosis and disease progression.

**Supplementary Information:**

The online version contains supplementary material available at 10.1007/s12350-021-02857-7.

## Introduction

Transthyretin cardiac amyloidosis (ATTR-CA) is an increasingly recognized, progressive, and fatal cardiomyopathy.^1^ Autopsy studies have found cardiac ATTR amyloid deposition in up to 25% of individuals over 80 years of age.^2^ Bone scintigraphy using ^99m^Technetium-pyrophosphate (^99m^Tc-PYP), ^99m^Tc-3,3-diphosphono-1,2-propanodicarboxylic acid, or ^99m^Tc-hydroxymethylene diphosphonate is a useful non-invasive examination for the diagnosis of ATTR-CA. A large multicenter study demonstrated that in patients who had undergone serum and urine laboratory tests to exclude light-chain cardiac amyloidosis, bone scintigraphy enables the diagnosis of ATTR-CA with high specificity without invasive biopsy.^3^

Because of the recent introduction of life-prolonging drugs for ATTR-CA, there is a major unmet need for the early detection and precise quantitative assessment of disease burden, progression, and treatment response.^4^ A multicenter study reported that planar heart-to-contralateral lung (H/CL) ratio of ^99m^Tc-PYP uptakes had prognostic information.^5^ However, the H/CL ratio is based on two-dimensional imaging and has some inherent limitations, such as influences of blood pool and rib uptakes.^6^ Volumetric evaluation of bone scintigraphy with single-photon emission computed tomography/computed tomography (SPECT/CT) is an objective and quantitative method to overcome weaknesses of conventional methods.^7,8^ The volume of the myocardial region with abnormal uptake is expected to assess disease burden and has a close association with cardiac functional parameters. However, the methodology and characteristics of volumetric parameters have not been fully investigated in patients with ATTR-CA. The aim of this study was to investigate the methodology of volumetric evaluation of ^99m^Tc-PYP SPECT/CT and to assess its correlation with cardiac functional parameters and other quantitative uptake parameters.

## Materials and Methods

### Study Population

We retrospectively evaluated patients who underwent ^99m^Tc-PYP SPECT/CT for suspected ATTR-CA at our hospital between October 2018 and June 2020. Among them, only patients who underwent endomyocardial biopsy (EMB) and/or TTR gene testing were included. Diagnosis criteria of ATTR-CA were based on one or more of the following: (1) EMB positive for ATTR or (2) documented TTR genetic mutation and evidence of cardiomyopathy without evidence of plasma cell dyscrasia (serum and urine immunofixation and a serum free light chain assay). For patients with multiple ^99m^Tc-PYP studies, only the first study was included. The study was approved by the institutional ethics committee of our university (2019-218) with waiver of the requirement for informed consent as a retrospective patient selection.

### Imaging Acquisition

Patients received 740 MBq (20 mCi) of ^99m^Tc-PYP intravenously, and thorax planar images were obtained at 3 hours after injection over a 2-minute duration on a hybrid SPECT/CT system (Symbia Intevo, Siemens Medical Solutions AG, Erlangen, Germany) using a low-energy high-resolution collimator. SPECT/CT images of the thorax were acquired at 3 hours after injection with the following parameters: step-and-shoot acquisition with body contour, 120 steps at 15 s/step, and zoom 1.0.

Images were reconstructed to a 128 × 128 matrix with a dedicated iterative algorithm (xSPECT QUANT) with 72 iterations and 1 subset, a 10-mm Gaussian filter. A low-dose non-contrast CT scan for attenuation correction was performed with the following parameters: 130 kV, 50 mAs with CARE Dose, pitch 1.5, rotation time 0.6 seconds, and collimation 16 × 1.2. The additional CT caused an estimated mean supplemental radiation dose of 1.0 mSv. Both the CT-based attenuation correction and a dual-energy scatter correction were automatically performed.

### Quantitative Imaging Interpretation

**Figure 1:**
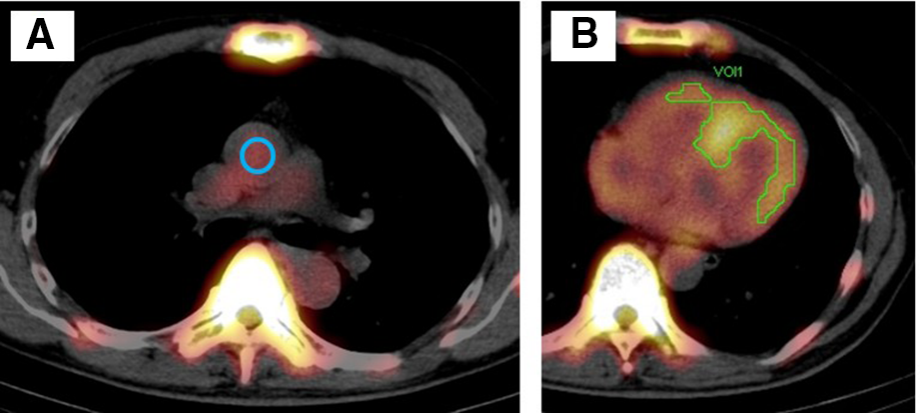
An example of SUV-thresholded volumetric evaluation. The ^99m^Technetium-pyrophosphate (^99m^Tc-PYP) activity was shown as an overlay on the computed tomographic imaging. A spherical volume of interest (blue circle, **a**) was placed in the center of the ascending aortic blood pool (ABP) at the height of the pulmonary artery bifurcation. The myocardial regions where ^99m^Tc-PYP uptakes were > 1.2 × ABP radioactivity (SUVmax) were automatically evaluated using xSPECT Quant (green contour, **b**). SUV, standardized uptake value

An example of SPECT/CT volumetric evaluation is shown in Figure 1. First, we measured ^99m^Tc-PYP activity in an aortic blood pool (ABP) using maximum standardized uptake value (SUVmax) and defined it as ABPmax. We placed a spherical volume of interest (VOI) in the center of the ascending aorta at the height of the pulmonary artery bifurcation using fused SPECT/CT images.^9^ The diameter of the VOI was half the size of the aortic diameter. Next, we placed a polygonal VOI manually to encompass the whole left and right ventricles, excluding adjacent rib and sternal uptakes. The total volumes of the myocardial regions where ^99m^Tc-PYP uptakes were > 1.2 and 1.4 × ABPmax were automatically evaluated and defined as cardiac pyrophosphate volume (CPV1.2 and CPV1.4: volume of voxels with abnormal ^99m^Tc-PYP uptakes).^7,8^ We used xSPECT Quant (Siemens) for calculating ABPmax and CPV. Because of the limitation of image resolution and the software, CPV less than 0.2 cm^3^ was rounded to 0.0 cm^3^. In patients with ATTR-CA, we also evaluated SUVmax within the whole left and right ventricular myocardium and the SUVmax was normalized to ABPmax (nSUVmax: SUVmax divided by ABPmax).^9,10^

As a conventional parameter, the H/CL ratio was also calculated as the counts in a circular region of interest (ROI) drawn over the heart of a planar image, which was divided by background counts in an identical size ROI over the contralateral chest.^6^ SPECT/CT images were also visually interpreted by experienced readers (SW and HW) blinded to clinical information and the results of the other imaging modality. The visual interpretation was graded qualitatively as positive (focal or diffuse uptake equal to or greater than rib uptake) or negative within the whole left and right ventricular myocardium.^11^

### Other Examinations

All patients with ATTR-CA (*n* = 14) underwent echocardiography 4.6 ± 5.0 days before ^99m^Tc-PYP imaging. Left ventricular ejection fraction (LVEF), left ventricular posterior wall thickness at end-diastole (LVPWTd), and E-to-early diastolic mitral annular tissue velocity ratio (*E*/*e*′) were assessed. The QRS duration on the electrocardiogram (ECG) was also measured 4.9 ± 2.9 days before ^99m^Tc-PYP imaging. In addition, b-type natriuretic peptide (BNP) was measured by a blood examination test 4.9 ± 2.7 days before ^99m^Tc-PYP imaging. All these parameters (LVEF, LVPWTd, *E*/*e*′, QRS, and BNP) were assessed in all 14 patients with ATTR-CA.

### Analyses

The primary analysis was to optimize the method of volumetric evaluation for diagnosing ATTR-CA by comparing thresholds (1.2 and 1.4 × ABPmax). The secondary analysis was the correlation of CPV with cardiac functional parameters of echocardiography (LVEF, LVPWTd, and *E*/*e*′), ECG (QRS), and blood examination (BNP). We also analyzed the correlation of CPV with other quantitative uptake parameters.

### Statistical Analysis

Continuous variables were summarized as mean ± standard deviation (SD) and compared with a student’s *t* test. Categorical variables were summarized as a number (percentage) and compared with a Chi-square or Fisher exact test as appropriate. Diagnostic accuracy for ATTR-CA was examined by receiver operating characteristic (ROC) analysis, and the area under the ROC curve (AUC) was calculated. Correlation of parameters was assessed with Pearson’s correlation coefficient. All statistical tests were two-sided, with *P* < .05 considered statistically significant. All analyses were performed using JMP version 12 (SAS Institute, Cary, NC, USA).

## Results

### Patient Population

In total, 25 patients were included with a mean age of 70.4 ± 12.8 years and 15 (60%) males. Population characteristics are summarized in Table 1. Fourteen (56%) patients were diagnosed with ATTR-CA, including hereditary type (ATTRv-CA, *n *= 9) and wild type (ATTRwt-CA, *n* = 5). Among patients diagnosed with ATTRv-CA, 8 (89%) patients had Val30Met [p.V50M] mutation and 1 (11%) patient had Leu58Arg [p.L78A] mutation. Eleven (44%) patients were diagnosed with no ATTR-CA and used as control patients. All control patients underwent EMB and were negative for ATTR.

**Table 1:** Population characteristics

	ATTR-CA (*n *= 14)	No ATTR-CA (*n *= 11)	*P* value
Age (years)	73.2 ± 13.0	66.8 ± 12.0	.21
Male	8 (57)	7 (64)	1.00
Types of ATTR-CA			
ATTRv-CA	9 (64)	–	–
ATTRwt-CA	5 (36)	–	
Diagnosis of no ATTR-CA patients			
Hypertrophic cardiomyopathy	–	3 (27)	–
Aortic stenosis	–	2 (18)	
AL-CA	–	1 (9)	
Cardiac sarcoidosis	–	1 (9)	
Dilated cardiomyopathy	–	1 (9)	
Hypertensive heart disease	–	1 (9)	
MGUS	–	1 (9)	
POEMS syndrome	–	1 (9)	
Types of examination			
Endomyocardial biopsy	8 (57)	11 (100)	–
TTR gene testing	14 (100)	1 (9)	

Continuous data are summarized as mean ± standard deviation, and categorical data as a number (percentage).

*AL-CA* light-chain cardiac amyloidosis, *ATTR-CA* transthyretin cardiac amyloidosis, *ATTRv* hereditary ATTR, *ATTRwt* wild-type ATTR, *MGUS* monoclonal gammopathy of undetermined significance, *POEMS* polyneuropathy, organomegaly, endocrinopathy, M protein, and skin changes, *TTR* transthyretin

### Quantitative ^99m^Tc-PYP Uptake Metrics

**Figure 2:**
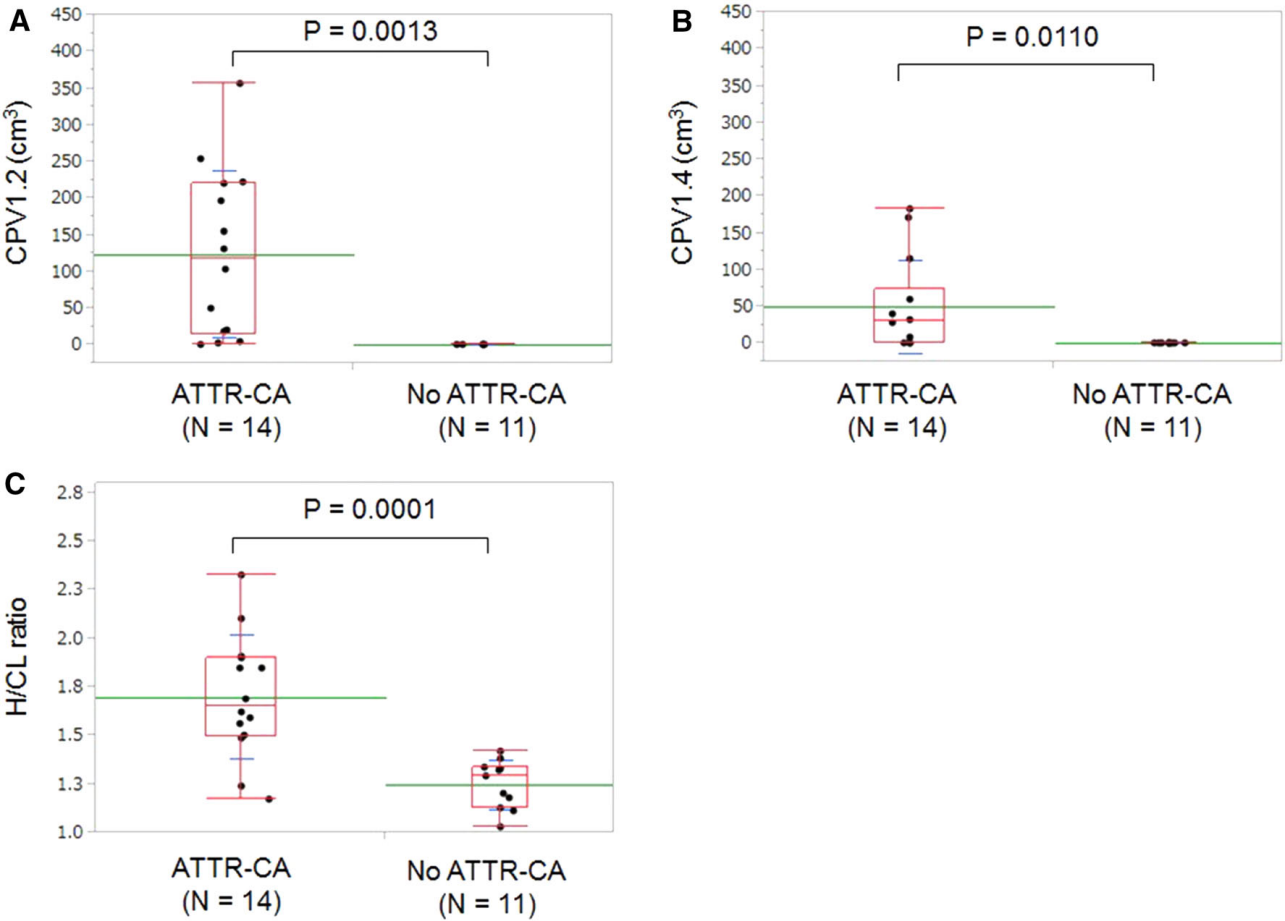
Comparison of CPV1.2, CPV1.4, and H/CL ratio between ATTR-CA patients and no ATTR-CA patients. CPV1.2 and CPV1.4 used 1.2 and 1.4 × aortic blood pool SUVmax as the SUV threshold. Green and blue lines are mean and standard deviation, respectively. Outlier box plot indicates median, 25%, and 75% quartile with whiskers for both ends. *ATTR-CA* transthyretin cardiac amyloidosis, *CPV* cardiac pyrophosphate volume, *H/CL* heart to contralateral lung, *SUV* standardized uptake value

CPV and H/CL ratios were significantly higher (*P* < .02) in patients with ATTR-CA than in those without ATTR-CA (Table 2 and Figure 2). In all patients with negative EMB for ATTR (*n *= 11), myocardial uptake was lower than ABPmax × 1.2 and CPV1.2 was 0.0 cm^3^. CPV1.2 showed no significant difference between patients with ATTRv-CA (113.5 ± 98.6 cm^3^, *n *= 9) and those with ATTRwt-CA (143.1 ± 148.2 cm^3^, *n* = 5).

**Table 2:** Quantitative ^99m^Technetium-pyrophosphate uptake metrics

	ATTR-CA (*n *= 14)	No ATTR-CA (*n *= 11)	*P* value
CPV1.2 (cm^3^)	124.0 ± 113.8	0.0 ± 0.0	.0013
CPV1.4 (cm^3^)	50.1 ± 63.3	0.0 ± 0.0	.0110
H/CL ratio	1.70 ± 0.32	1.25 ± 0.13	.0001
SUVmax	3.00 ± 0.86	–	–
nSUVmax	1.70 ± 0.43	–	–

Continuous variables are displayed as mean ± standard deviation. SUVmax and nSUVmax were not evaluated in control patients because their myocardial abnormal uptakes were absent and their adjacent blood pool radioactivity was relatively high.

*ATTR-CA* transthyretin cardiac amyloidosis, *CPV* cardiac pyrophosphate volume, *H/CL* heart to contralateral lung, *nSUV* normalized SUV, *SUV* standardized uptake value

### Diagnostic Accuracy

**Figure 3:**
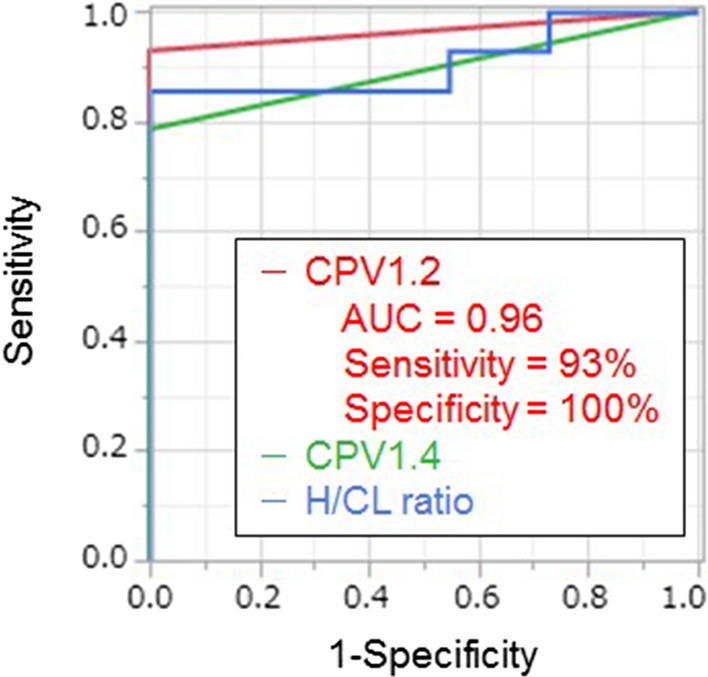
ROC curves of CPV1.2, CPV1.4, and H/CL ratio for identifying transthyretin cardiac amyloidosis. *AUC* area under receiver operating characteristic curve, *CPV* cardiac pyrophosphate volume, *H/CL* heart to contralateral lung

ROC curves for identifying ATTR-CA are summarized in Figure 3. Diagnostic accuracy of uptake parameters is summarized in Table 3. CPV1.2 achieved the highest AUC, sensitivity, and specificity of 0.96, 93%, and 100%, respectively. Two patients with EMB-proven ATTRwt-CA were correctly diagnosed with CPV1.2, whereas CPV1.4 misdiagnosed them as negative because their myocardial nSUVmax were 1.34 and 1.40 and abnormal uptakes were focal. One EMB-proven ATTRv-CA patient with Val30Met mutation was misdiagnosed by each uptake parameters and even visual SPECT/CT evaluation because abnormal myocardial uptake was absent. SPECT/CT images of these three ATTR-CA patients are shown in Supplemental Figure S1. AUC of CPV1.2, CPV1.4, and H/CL ratio was 0.96, 0.89, and 0.91, respectively. The AUC showed no significant difference among parameters. CPV1.2 and visual SPECT/CT interpretation showed the same diagnostic accuracy for ATTR-CA (Table 3).

**Table 3:** Diagnostic accuracy for transthyretin cardiac amyloidosis

	AUC	Threshold	Sensitivity (%)	Specificity (%)	PPV (%)	NPV (%)
CPV1.2	0.96	2.62	93	100	100	92
CPV1.4	0.89	0.33	79	100	100	79
H/CL ratio	0.91	1.49	86	100	100	85
Visual SPECT/CT interpretation	–	–	93	100	100	92

*AUC* area under receiver operating characteristic curve, *CPV* cardiac pyrophosphate volume, *H/CL* heart to contralateral lung, *NPV* negative predictive value, *PPV* positive predictive value, *SPECT* single-photon emission computed tomography

### Correlation with Cardiac Functional Parameters

**Figure 4:**
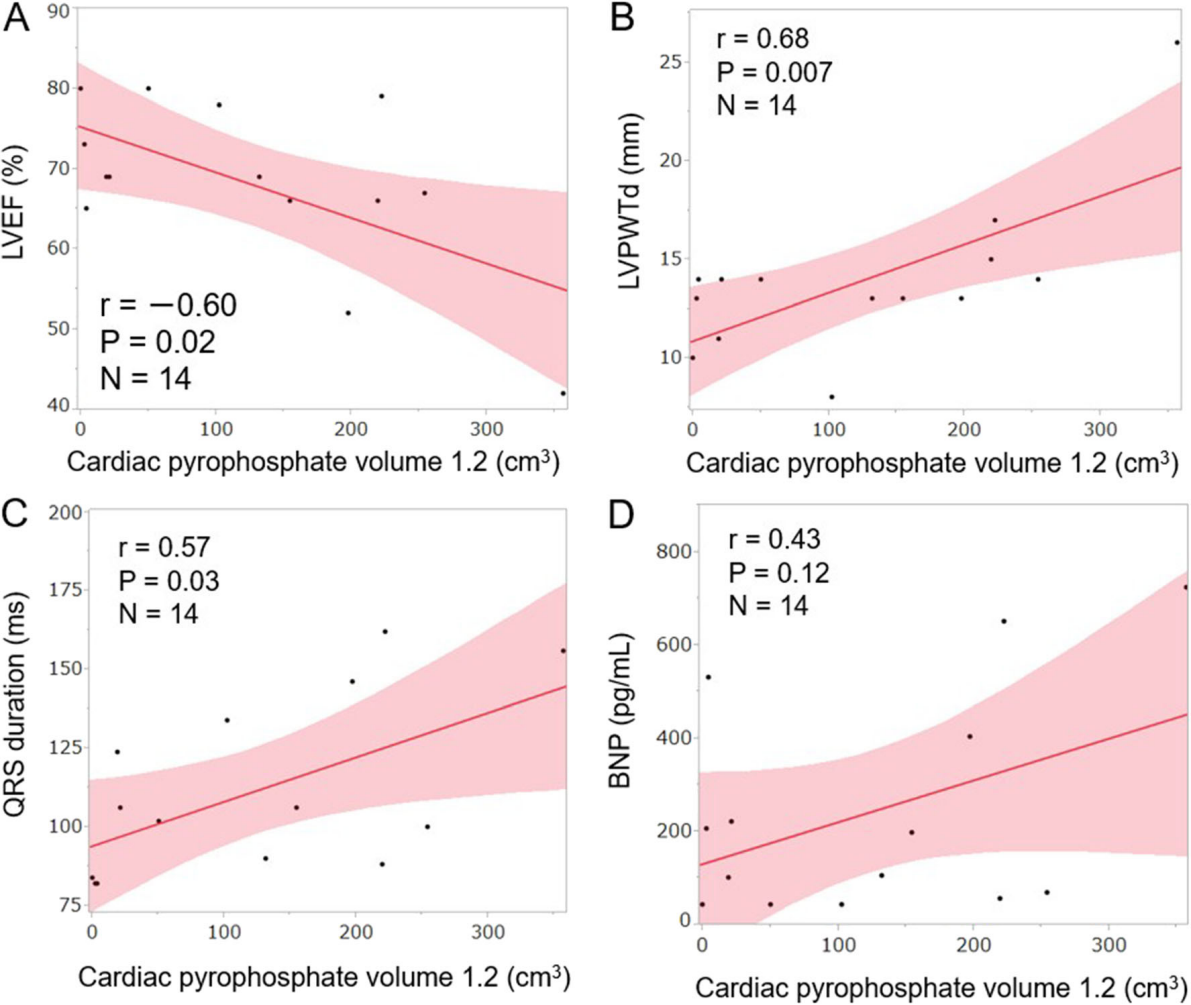
Correlation between CPV1.2 and various cardiac parameters in patients with transthyretin cardiac amyloidosis. Solid lines indicate predicted values from linear regression analysis with the shaded band showing the 95% CI. *BNP* b-type natriuretic peptide, *CPV* cardiac pyrophosphate volume, *LVEF* left ventricular ejection fraction, *LVPWTd* left ventricular posterior wall thickness at end-diastole

The correlation between uptake parameters and cardiac functional parameters in patients with ATTR-CA is summarized in Table 4 and Figure 4. CPV1.2 moderately and negatively correlated with LVEF (mean ± SD = 68.2 ± 10.7%) and moderately and positively correlated with LVPWTd (13.9 ± 4.1 mm), QRS (111.6 ± 28.0 ms), and BNP (242.5 ± 237.8 pg/mL). Correlation between CPV1.2 and *E*/*e*′ (18.4 ± 5.1) was low. CPV1.2 significantly correlated with LVEF, LVPWTd, and QRS. The H/CL ratio did not significantly correlate with any of the values of LVEF, LVPWTd, *E*/*e*′, QRS, and BNP.

**Table 4:** Correlation between uptake parameters and cardiac functional parameters

	CPV1.2		H/CL ratio		SUVmax	
	*r*	*P* value	*r*	*P* value	*r*	*P* value
LVEF	− 0.60	.02*	− 0.14	.63	− 0.55	.04*
LVPWTd	0.68	.007*	0.37	.20	0.32	.27
*E*/*e*′	0.16	.58	0.19	.52	0.11	.70
QRS	0.57	.03*	0.35	.22	0.43	.13
BNP	0.43	.12	0.28	.33	0.12	.67

Correlation in patients diagnosed with ATTR-CA (n = 14).

*ATTR-CA* transthyretin cardiac amyloidosis, *BNP* b-type natriuretic peptide, *CPV* cardiac pyrophosphate volume, *E/e’* E-to-early diastolic mitral annular tissue velocity ratio, *H/CL* heart to contralateral lung, *LVEF* left ventricular ejection fraction, *LVPWTd* left ventricular posterior wall thickness at end-diastole, *r* Pearson’s correlation coefficient, *SUV* standardized uptake value

*P < .05.

### SUVmax

In one EMB-proven ATTRv-CA patient who did not show abnormal myocardial activity, we evaluated SUVmax in the interventricular septum using fused SPECT/CT images. Fourteen patients with ATTR-CA showed negative correlation between SUVmax and LVEF, but the SUVmax did not significantly correlate with other parameters (LVPWTd, *E*/*e*′, QRS, and BNP).

### Correlation with Other Quantitative PYP Uptake Parameters

**Figure 5:**
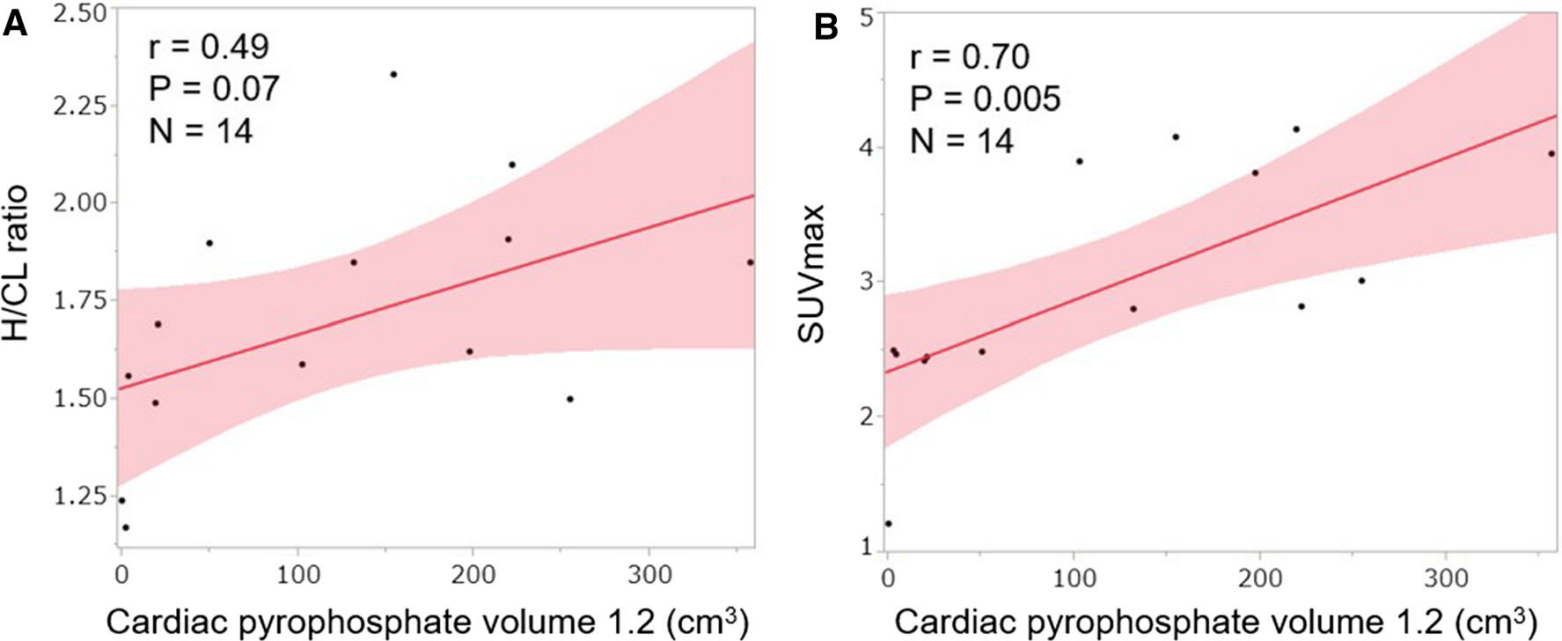
Correlation between CPV1.2 and various uptake parameters in patients with transthyretin cardiac amyloidosis. Solid lines indicate predicted values from linear regression analysis with the shaded band showing the 95% CI. *CPV* cardiac pyrophosphate volume, *H/CL* heart to contralateral lung, *SUV* standardized uptake value

In patients with ATTR-CA, CPV1.2 moderately correlated with the H/CL ratio (*r *= 0.49, Figure 5a) and strongly correlated with SUVmax (*r *= 0.70, Figure 5b).

## Discussion

This study suggests two important points. First, CPV1.2 was superior to CPV1.4 and the H/CL ratio in diagnosing ATTR-CA. Second, CPV1.2 was superior to the H/CL ratio and SUVmax in its correlation with cardiac functional parameters.

Our results demonstrated that setting the myocardial ^99m^Tc-PYP uptake threshold at 1.2 × ABPmax was optimal for detecting focal abnormal uptakes. If focal ATTR amyloid deposition in patients with early-stage ATTR-CA can be detected early, new life-prolonging drugs would be of greater benefit.^4^ Although Miller et al used a threshold, determined as 1.5 × left ventricular blood pool (LVBP) ^99m^Tc-PYP activity in SPECT, their method was based on previous studies of ^18^F-fluorodeoxyglucose positron emission tomography with suspected cardiac sarcoidosis patients.^7^ As shown by our results of CPV1.4, higher thresholds can cause false-negative diagnosis in patients with focal abnormal uptakes. Given the high diagnostic accuracy of visual SPECT/CT interpretation, it is unlikely that volumetric evaluation could improve diagnostic accuracy. However, it may have a role as a quantitative and objective marker.

Volumetric evaluation of bone scintigraphy can be an objective marker for disease burden of ATTR-CA and provide valuable prognostic information. We demonstrated that CPV1.2 significantly correlated with several cardiac functional parameters (LVEF, LVPWTd, and QRS). In addition, the significant correlation was stronger in CPV1.2 than in the H/CL ratio and SUVmax. Although CPV1.2 did not significantly correlate with BNP, the correlation was stronger in CPV1.2 than in the H/CL ratio and SUVmax. The H/CL ratio is based on two-dimensional imaging and has some inherent limitations. Reduced LVEF < 50% was reported as a predictor of mortality in patients with ATTRwt-CA (hazard ratio: 1.85).^12^ N-terminal pro-BNP (NT-proBNP) and the estimated glomerular filtration rate were reported to stratify patients with both ATTRwt-CA and ATTRv-CA into prognostic categories.^13^ NT-proBNP and high-sensitivity cardiac troponin T were reported to assess prognosis in patients with ATTRwt-CA.^12^ Therefore, we expect CPV to play an important role in risk assessment. Miller et al also demonstrated that their volumetric parameters of ^99m^Tc-PYP SPECT had prognostic value and low inter-observer variability.^7^

We included only patients with negative EMB for ATTR as control patients, which is a unique and novel feature of our study. Several studies adopted positive standards for ATTR-CA as follows: (1) visual grade of 2 or 3 for myocardial uptake (equal to or more than that of rib uptake), (2) diffuse abnormal myocardial uptake in SPECT.^14,15^ However, some patients with ATTR-CA showed focal or absent ^99m^Tc-PYP uptakes.^3,16,17^ We found several patients who had positive results of EMB and their myocardial ^99m^Tc-PYP uptakes were focal (*n *= 2) or absent (*n *= 1). Sperry et al also reported that 1 of 33 (3%) patients with myocardial ^99m^Tc-PYP uptakes had focal uptake.^11^ In addition, several causes of false-negative and false-positive results of bone scintigraphy in patients with suspected ATTR-CA have been reported, including Val30Met mutation.^18^

The method of placing a reference VOI in the ascending aorta at the height of the pulmonary artery bifurcation is objective to evaluate background radiotracer activity.^9^ Sperry et al also calculated mean blood pool counts at the ascending aorta.^16^ In addition, variation of radioactivity was reported to be relatively smaller in the aorta than in bone.^9^ Bone radioactivity has often been used as a reference for myocardial uptake.^6,10^ However, the method of placing a reference VOI in the LVBP^7^ can be subjective. In addition, in patients with small hearts, their LVBP activity can be contaminated by the spillover of myocardial uptake.

SUVmax has some inherent limitations and we did not evaluate it in patients without ATTR-CA. SUV depends on VOI definition, and given the poor spatial resolution of SPECT, it is often difficult to discriminate between relatively high blood pool radioactivity and uptake in the adjacent myocardial wall, especially in normal and equivocal patients.^9,14^ Due to cardiac motion from contraction and breathing, the position of the heart shown on a CT scan is not perfectly coregistered to the SPECT images. On a non-contrast low-dose CT scan, which is used in SPECT/CT, the myocardium is poorly visualized in normal patients; hence, defining a VOI which covers only myocardium is difficult in normal hearts. In addition, SUVmax represents only a single voxel value and can be contaminated by spillover from neighboring high bone activity.

## Limitations

Our small population and single-center study design are weakness of this study. The thresholds and diagnostic accuracy of uptake parameters were based on our small datasets. The present findings require larger, multicenter, prospective validations. Our method needs a manual setting of a polygonal VOI which encompasses the left and right ventricles based on fused SPECT/CT images. However, if we use a proper uptake threshold, the influence of VOI variation is limited. We included only patients with negative EMB for ATTR as control patients, and the selection may have caused a bias. In daily practice, not all suspected ATTR-CA patients undergo EMB.

## Conclusion

Optimized volumetric evaluation of ^99m^Tc-PYP SPECT/CT may be superior to the H/CL ratio and SUVmax in diagnosing patients with ATTR-CA and assessing the disease burden. Larger studies are warranted to clarify whether volumetric measurement of bone scintigraphy can identify patients with early-stage ATTR-CA and assess prognosis, disease progression, and treatment response.

## New Knowledge Gained

Volumetric evaluation of ^99m^Tc-PYP SPECT/CT was useful for detecting both focal and diffuse abnormal uptakes and showed a high diagnostic accuracy for ATTR-CA. Cardiac pyrophosphate volume was superior to the H/CL ratio and SUVmax in its correlation with cardiac functional parameters and may be a useful non-invasive marker for assessing the disease burden of ATTR-CA.

## Supplementary Information

Below is the link to the electronic supplementary material.

SPECT/CT images of three EMB-proven ATTR-CA patients whose CPV were low. Their CPV1.2 were 3.9, 2.6, and 0.0 cm^3^ and their CPV1.4 were 0.0 cm^3^. The myocardial regions where ^99m^Technetium-pyrophosphate uptakes were > 1.2 × aortic blood pool SUVmax are shown by green contours in two patients (A and B). ATTR-CA, transthyretin cardiac amyloidosis; CPV, cardiac pyrophosphate volume; EMB, Endomyocardial biopsy; SPECT, Single-photon emission computed tomography; SUV, standardized uptake value. Supplementary file1 (PDF 83 kb)

Supplementary file2 (PPTX 453 kb)

## Abbreviations

ABP: Aortic blood pool
ATTR-CA: Transthyretin cardiac amyloidosis
CPV: Cardiac pyrophosphate volume
EMB: Endomyocardial biopsy
H/CL: Heart to contralateral lung
LVBP: Left ventricular blood pool
PYP: Pyrophosphate
SPECT: Single-photon emission computed tomography
SUV: Standardized uptake value
VOI: Volume of interest
