# Supplementary material for: Volumetric evaluation of 99mTc-pyrophosphate SPECT/CT for transthyretin cardiac amyloidosis: Methodology and correlation with cardiac functional parameters
Source: J Nucl Cardiol. 2021 Dec 14;29(6):3102–10. doi: 10.1007/s12350-021-02857-7 (PMC9834362; doi:10.1007/s12350-021-02857-7)
Supplement: Supplementary file 1 — SPECT/CT images of three EMB-proven ATTR-CA patients whose CPV were low. Their CPV1.2 were 3.9, 2.6, and 0.0 cm3 and their CPV1.4 were 0.0 cm3. The myocardial regions where 99mTechnetium-pyrophosphate uptakes were > 1.2 × aortic blood pool SUVmax are shown by green contours in two patients (A and B). ATTR-CA, transthyretin cardiac amyloidosis; CPV, cardiac pyrophosphate volume; EMB, Endomyocardial biopsy; SPECT, Single-photon emission computed tomography; SUV, standardized uptake value. Supplementary file1 (PDF 83 kb) [file 12350_2021_2857_MOESM1_ESM.pdf]

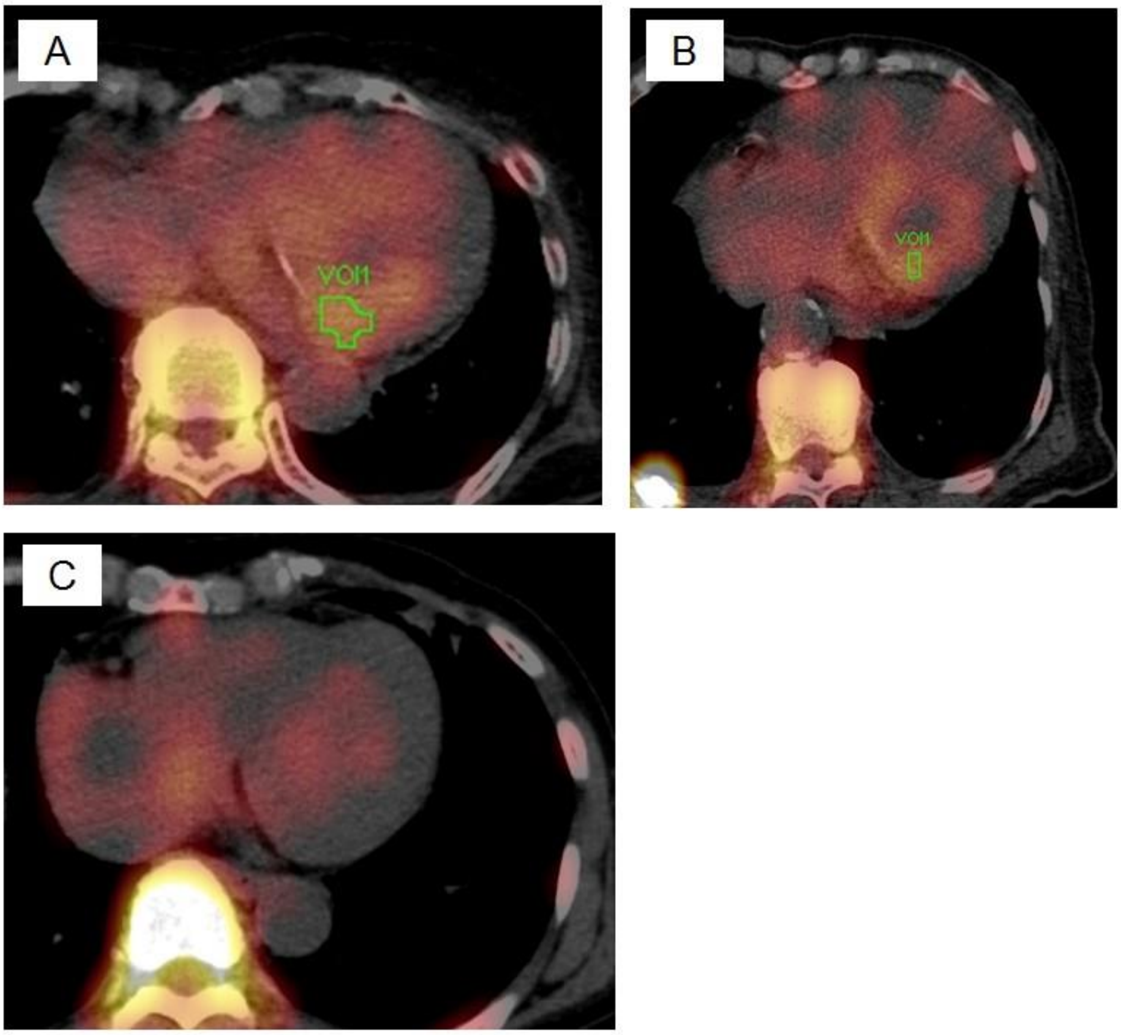

Supplemental Figure S1. SPECT/CT images of three EMB-proven ATTR-CA patients whose CPV were low. Their CPV1.2 were 3.9, 2.6, and 0.0 cm^3, and their CPV1.4 were 0.0 cm^3. The myocardial regions where ^{99m}Technetium-pyrophosphate uptakes were > 1.2 × aortic blood pool SUVmax are shown by green contours in two patients (A and B). *ATTR-CA* transthyretin cardiac amyloidosis, *CPV* cardiac pyrophosphate volume, *EMB* endomyocardial biopsy, *SPECT* single-photon emission computed tomography, *SUV* standardized uptake value.
