# Supplementary material for: Volumetric evaluation of 99mTc-pyrophosphate SPECT/CT for transthyretin cardiac amyloidosis: Methodology and correlation with cardiac functional parameters
Source: J Nucl Cardiol. 2021 Dec 14;29(6):3102–10. doi: 10.1007/s12350-021-02857-7 (PMC9834362; doi:10.1007/s12350-021-02857-7)
Supplement: Supplementary file 2 — Supplementary file2 (PPTX 453 kb) [file 12350_2021_2857_MOESM2_ESM.pptx]

## Slide 1
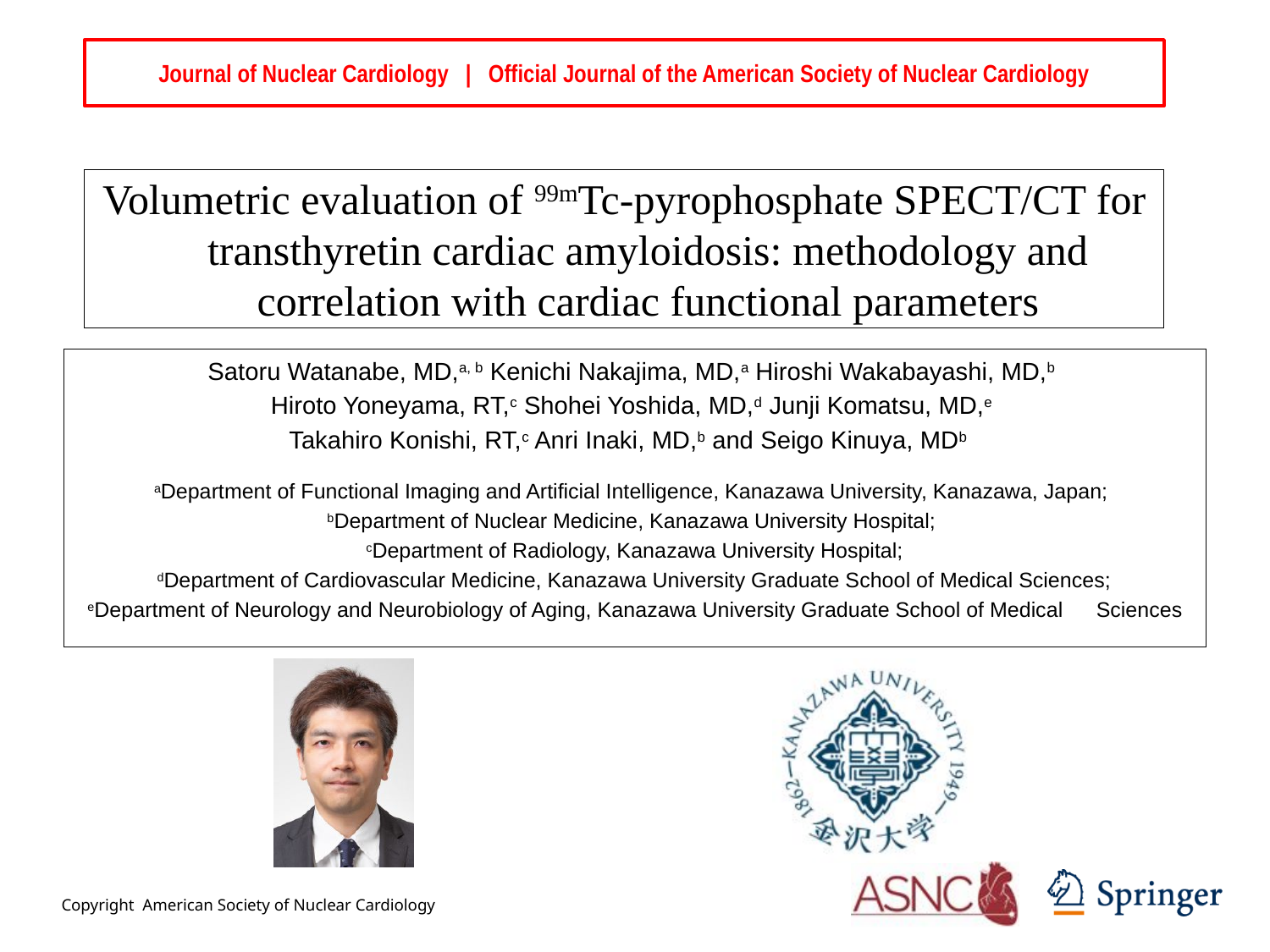

Journal of Nuclear Cardiology | Official Journal of the American Society of Nuclear Cardiology
# Volumetric evaluation of 99mTc-pyrophosphate SPECT/CT for transthyretin cardiac amyloidosis: methodology and correlation with cardiac functional parameters
Satoru Watanabe, MD,a, b Kenichi Nakajima, MD,a Hiroshi Wakabayashi, MD,b
Hiroto Yoneyama, RT,c Shohei Yoshida, MD,d Junji Komatsu, MD,e
Takahiro Konishi, RT,c Anri Inaki, MD,b and Seigo Kinuya, MDb
aDepartment of Functional Imaging and Artificial Intelligence, Kanazawa University, Kanazawa, Japan;
bDepartment of Nuclear Medicine, Kanazawa University Hospital;
cDepartment of Radiology, Kanazawa University Hospital;
 dDepartment of Cardiovascular Medicine, Kanazawa University Graduate School of Medical Sciences;
eDepartment of Neurology and Neurobiology of Aging, Kanazawa University Graduate School of Medical　Sciences
Copyright American Society of Nuclear Cardiology

## Slide 2
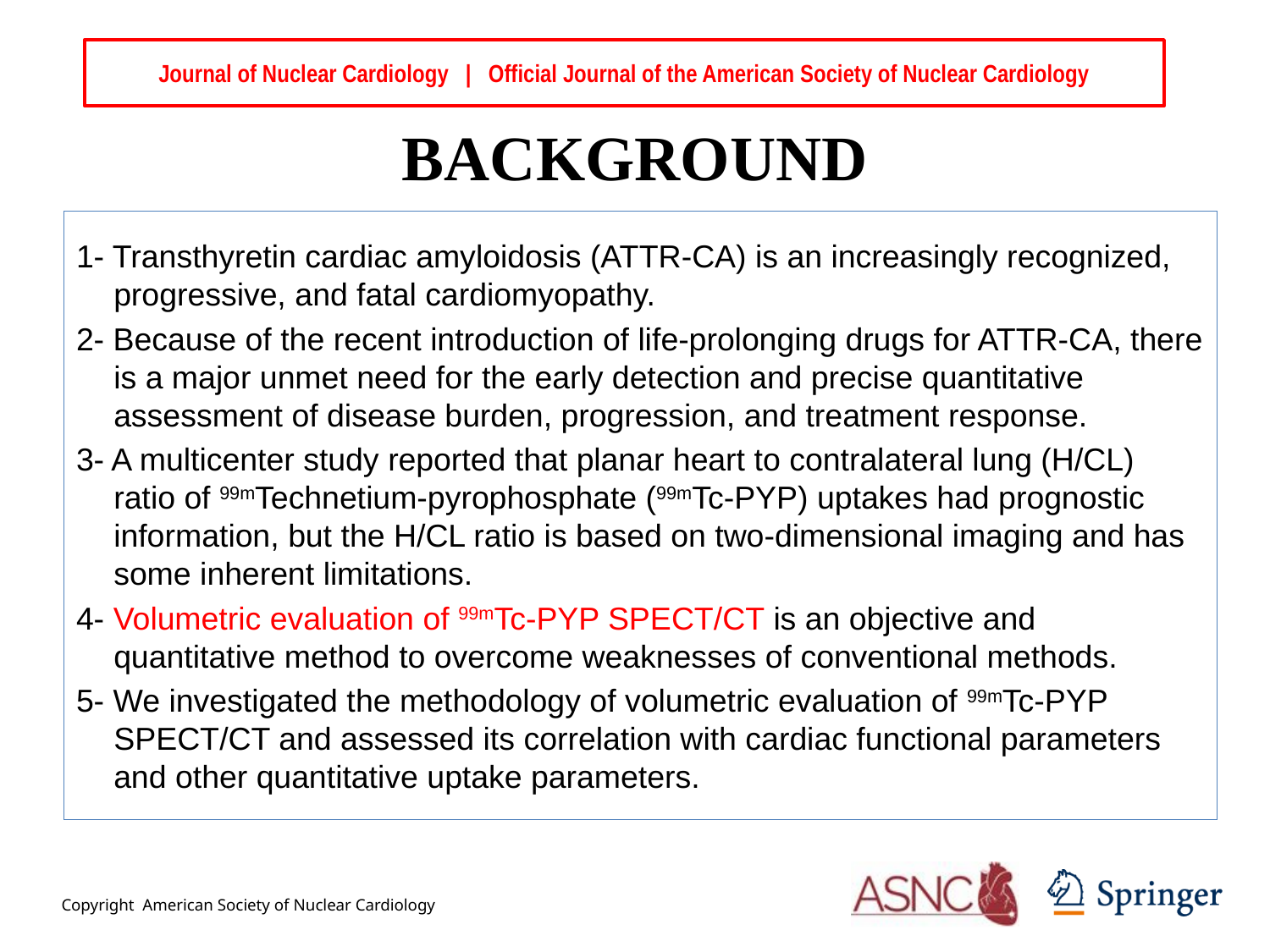

Journal of Nuclear Cardiology | Official Journal of the American Society of Nuclear Cardiology
# BACKGROUND
1- Transthyretin cardiac amyloidosis (ATTR-CA) is an increasingly recognized, progressive, and fatal cardiomyopathy.
2- Because of the recent introduction of life-prolonging drugs for ATTR-CA, there is a major unmet need for the early detection and precise quantitative assessment of disease burden, progression, and treatment response.
3- A multicenter study reported that planar heart to contralateral lung (H/CL) ratio of 99mTechnetium-pyrophosphate (99mTc-PYP) uptakes had prognostic information, but the H/CL ratio is based on two-dimensional imaging and has some inherent limitations.
4- Volumetric evaluation of 99mTc-PYP SPECT/CT is an objective and quantitative method to overcome weaknesses of conventional methods.
5- We investigated the methodology of volumetric evaluation of 99mTc-PYP SPECT/CT and assessed its correlation with cardiac functional parameters and other quantitative uptake parameters.
Copyright American Society of Nuclear Cardiology

## Slide 3
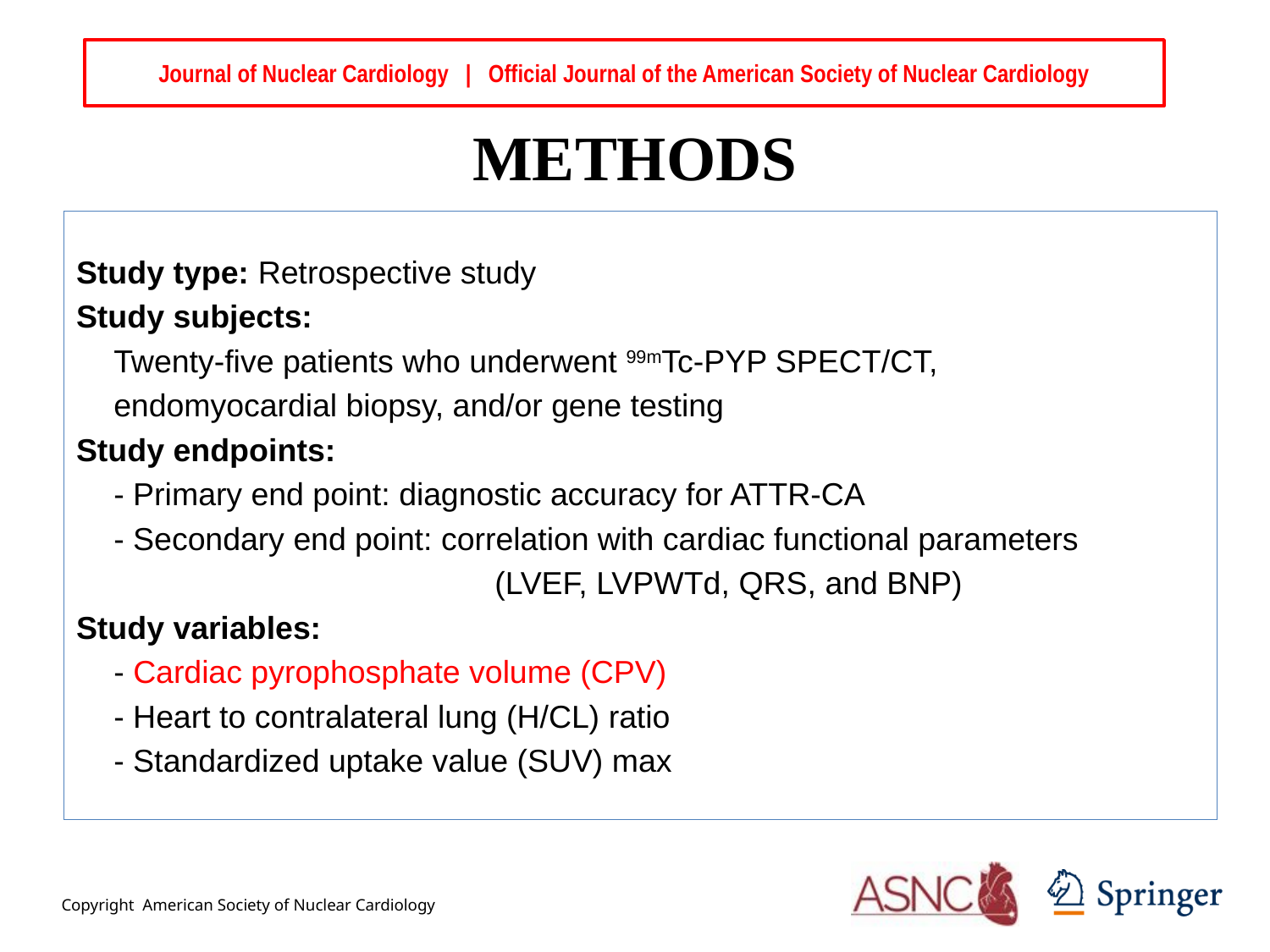

Journal of Nuclear Cardiology | Official Journal of the American Society of Nuclear Cardiology
# METHODS
Study type: Retrospective study
Study subjects:
	Twenty-five patients who underwent 99mTc-PYP SPECT/CT,
	endomyocardial biopsy, and/or gene testing
Study endpoints:
	- Primary end point: diagnostic accuracy for ATTR-CA
	- Secondary end point: correlation with cardiac functional parameters
				(LVEF, LVPWTd, QRS, and BNP)
Study variables:
	- Cardiac pyrophosphate volume (CPV)
	- Heart to contralateral lung (H/CL) ratio
	- Standardized uptake value (SUV) max
Copyright American Society of Nuclear Cardiology

## Slide 4
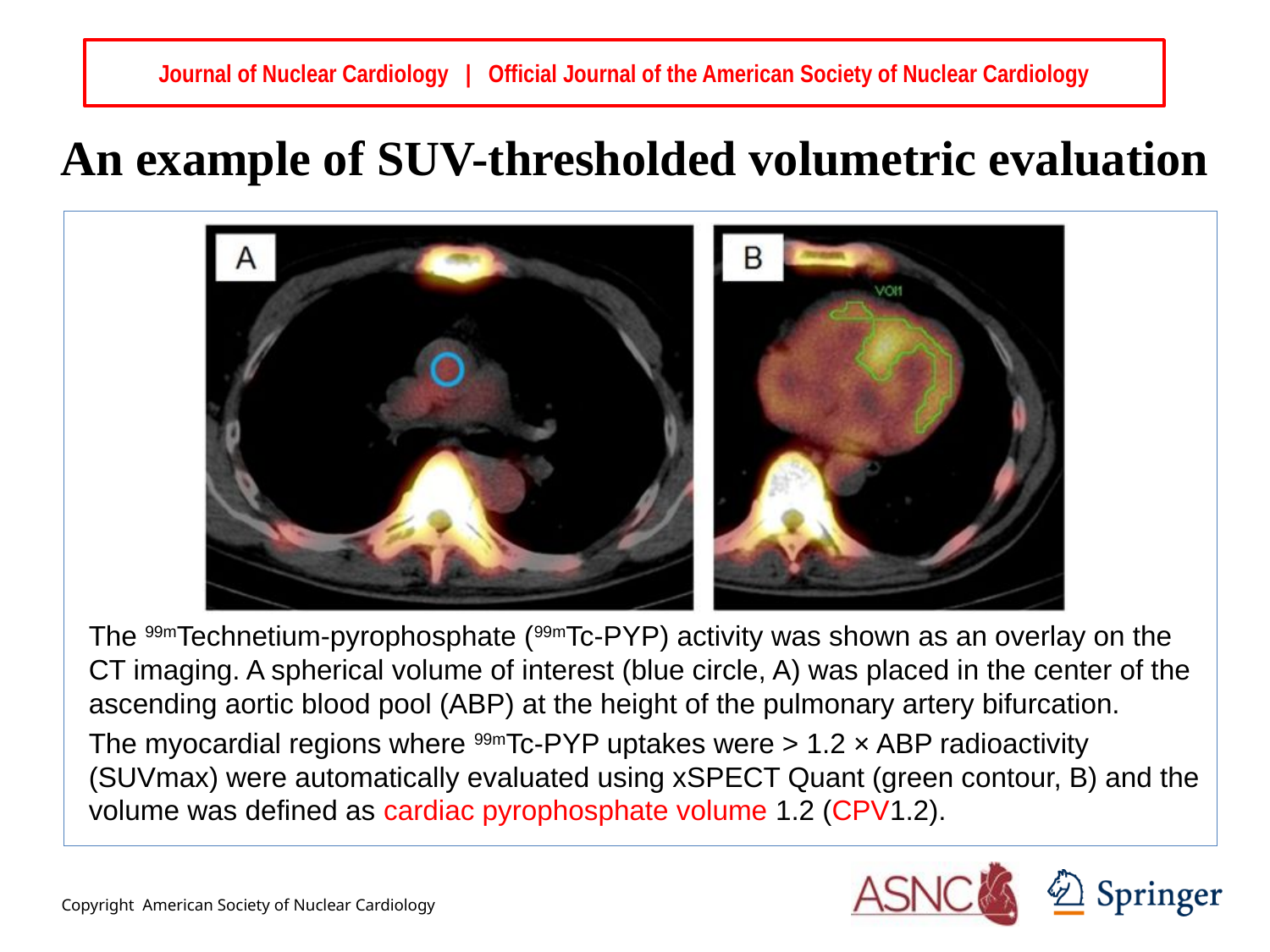

Journal of Nuclear Cardiology | Official Journal of the American Society of Nuclear Cardiology
# An example of SUV-thresholded volumetric evaluation
The 99mTechnetium-pyrophosphate (99mTc-PYP) activity was shown as an overlay on the CT imaging. A spherical volume of interest (blue circle, A) was placed in the center of the ascending aortic blood pool (ABP) at the height of the pulmonary artery bifurcation.
The myocardial regions where 99mTc-PYP uptakes were > 1.2 × ABP radioactivity (SUVmax) were automatically evaluated using xSPECT Quant (green contour, B) and the volume was defined as cardiac pyrophosphate volume 1.2 (CPV1.2).
Copyright American Society of Nuclear Cardiology

## Slide 5
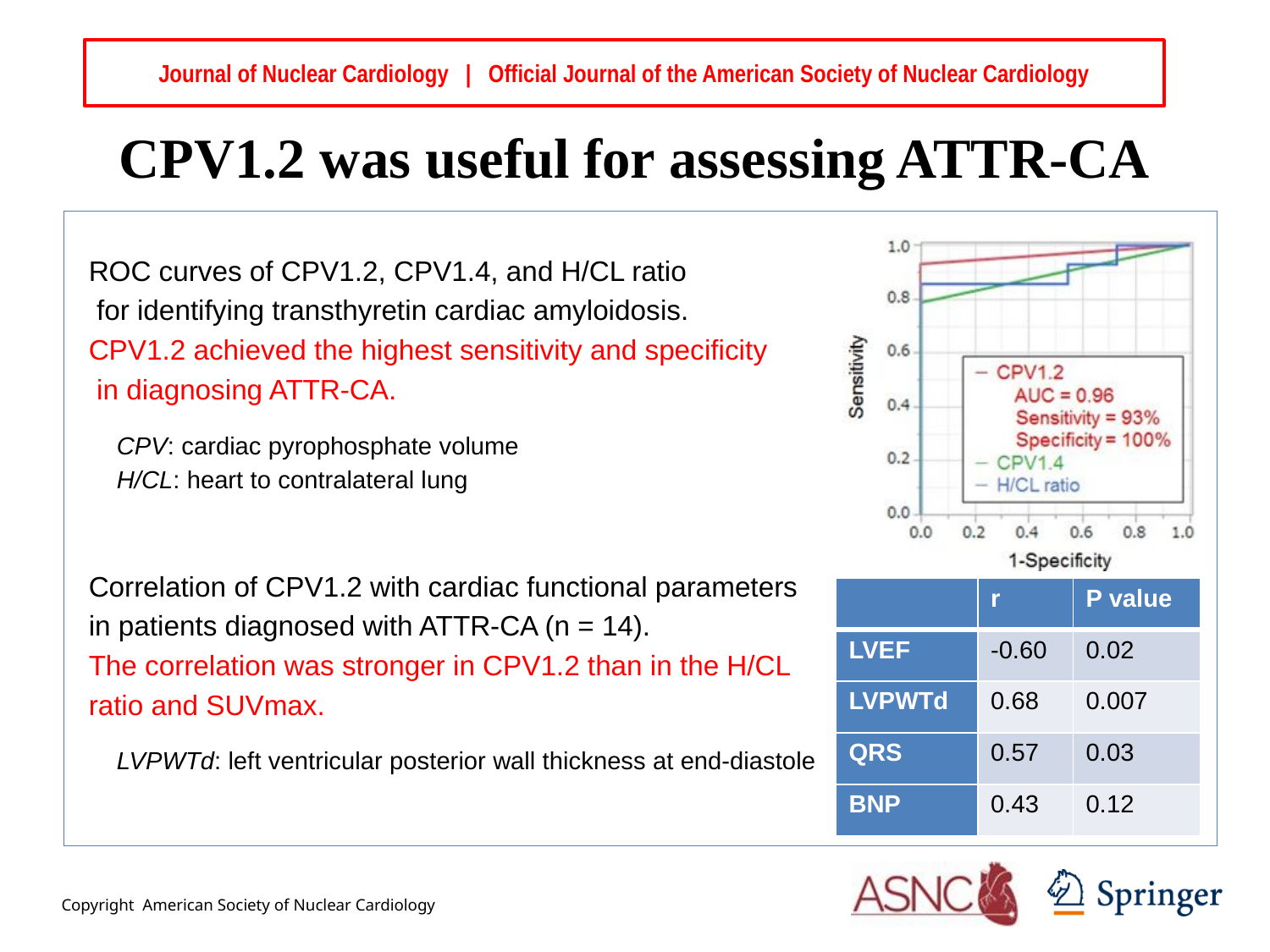

Journal of Nuclear Cardiology | Official Journal of the American Society of Nuclear Cardiology
# CPV1.2 was useful for assessing ATTR-CA
ROC curves of CPV1.2, CPV1.4, and H/CL ratio
 for identifying transthyretin cardiac amyloidosis.
CPV1.2 achieved the highest sensitivity and specificity
 in diagnosing ATTR-CA.
 CPV: cardiac pyrophosphate volume
 H/CL: heart to contralateral lung
Correlation of CPV1.2 with cardiac functional parameters
in patients diagnosed with ATTR-CA (n = 14).
The correlation was stronger in CPV1.2 than in the H/CL
ratio and SUVmax.
 LVPWTd: left ventricular posterior wall thickness at end-diastole
| | r | P value |
| --- | --- | --- |
| LVEF | -0.60 | 0.02 |
| LVPWTd | 0.68 | 0.007 |
| QRS | 0.57 | 0.03 |
| BNP | 0.43 | 0.12 |
Copyright American Society of Nuclear Cardiology

## Slide 6
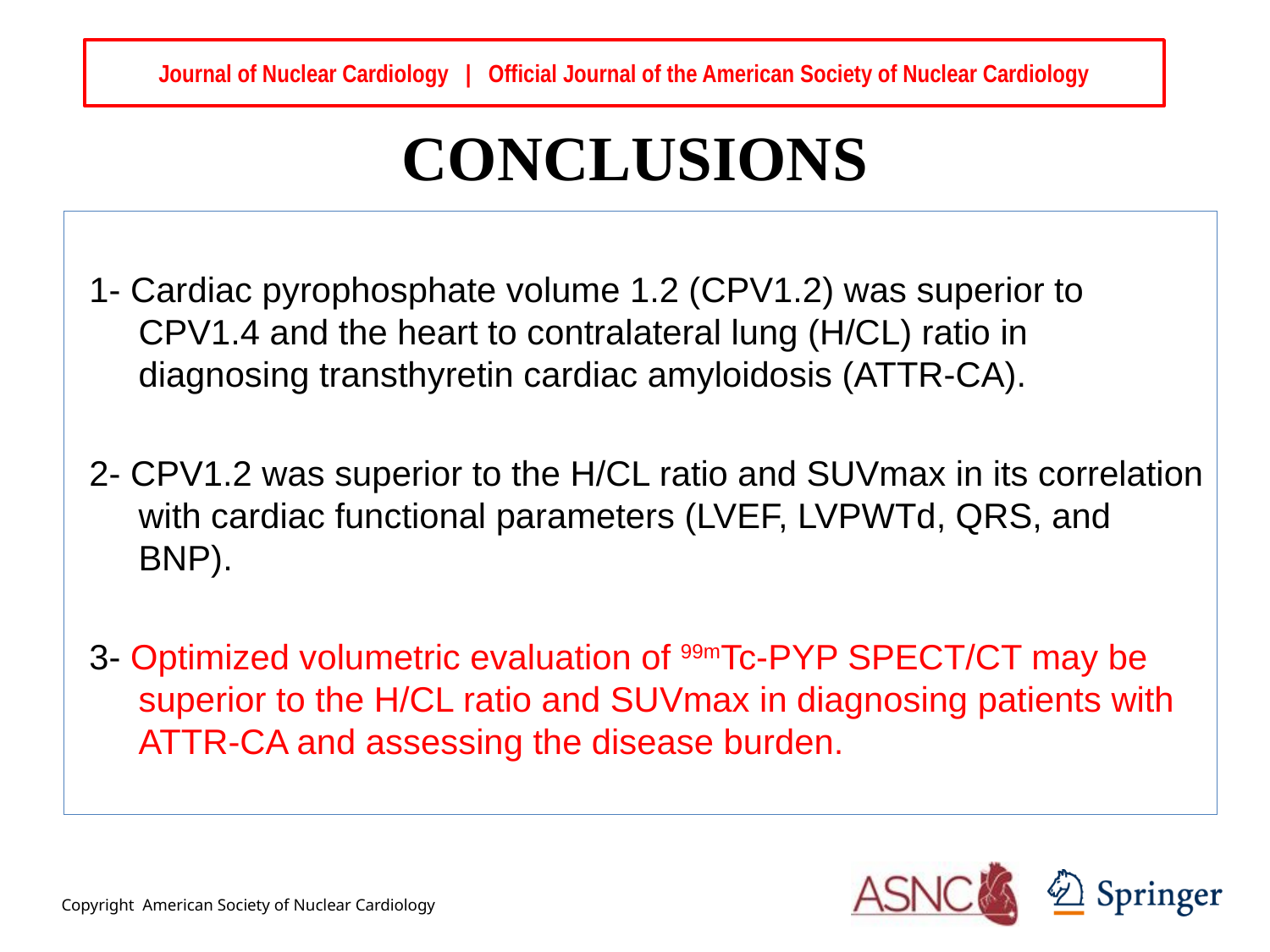

Journal of Nuclear Cardiology | Official Journal of the American Society of Nuclear Cardiology
# CONCLUSIONS
1- Cardiac pyrophosphate volume 1.2 (CPV1.2) was superior to CPV1.4 and the heart to contralateral lung (H/CL) ratio in diagnosing transthyretin cardiac amyloidosis (ATTR-CA).
2- CPV1.2 was superior to the H/CL ratio and SUVmax in its correlation with cardiac functional parameters (LVEF, LVPWTd, QRS, and BNP).
3- Optimized volumetric evaluation of 99mTc-PYP SPECT/CT may be superior to the H/CL ratio and SUVmax in diagnosing patients with ATTR-CA and assessing the disease burden.
Copyright American Society of Nuclear Cardiology
